# EBNA3C Directs Recruitment of RBPJ (CBF1) to Chromatin during the Process of Gene Repression in EBV Infected B Cells

**DOI:** 10.1371/journal.ppat.1005383

**Published:** 2016-01-11

**Authors:** Jens S. Kalchschmidt, Adam C. T. Gillman, Kostas Paschos, Quentin Bazot, Bettina Kempkes, Martin J. Allday

**Affiliations:** 1 Molecular Virology, Department of Medicine, Imperial College London, London, United Kingdom; 2 Department of Gene Vectors, Hematologikum, Helmholtz Zentrum München, National Research Center for Environmental Health, München, Germany; Tulane Health Sciences Center, UNITED STATES

## Abstract

It is well established that Epstein-Barr virus nuclear antigen 3C (EBNA3C) can act as a potent repressor of gene expression, but little is known about the sequence of events occurring during the repression process. To explore further the role of EBNA3C in gene repression–particularly in relation to histone modifications and cell factors involved–the three host genes previously reported as most robustly repressed by EBNA3C were investigated. *COBLL1*, a gene of unknown function, is regulated by EBNA3C alone and the two co-regulated disintegrin/metalloproteases, *ADAM28* and *ADAMDEC1* have been described previously as targets of both EBNA3A and EBNA3C. For the first time, EBNA3C was here shown to be the main regulator of all three genes early after infection of primary B cells. Using various EBV-recombinants, repression over orders of magnitude was seen only when EBNA3C was expressed. Unexpectedly, full repression was not achieved until 30 days after infection. This was accurately reproduced in established LCLs carrying EBV-recombinants conditional for EBNA3C function, demonstrating the utility of the conditional system to replicate events early after infection. Using this system, detailed chromatin immunoprecipitation analysis revealed that the initial repression was associated with loss of activation-associated histone modifications (H3K9ac, H3K27ac and H3K4me3) and was independent of recruitment of polycomb proteins and deposition of the repressive H3K27me3 modification, which were only observed later in repression. Most remarkable, and in contrast to current models of RBPJ in repression, was the observation that this DNA-binding factor accumulated at the EBNA3C-binding sites only when EBNA3C was functional. Transient reporter assays indicated that repression of these genes was dependent on the interaction between EBNA3C and RBPJ. This was confirmed with a novel EBV-recombinant encoding a mutant of EBNA3C unable to bind RBPJ, by showing this virus was incapable of repressing *COBLL1* or *ADAM28/ADAMDEC1* in newly infected primary B cells.

## Author Summary

The Epstein-Barr nuclear protein EBNA3C is a well-characterised repressor of host gene expression in B cells growth-transformed by EBV. It is also well established that EBNA3C can interact with the cellular factor RBPJ, a DNA-binding factor in the Notch signalling pathway conserved from worms to humans. However, prior to this study, little was known about the role of the interaction between these two proteins during the repression of host genes. We therefore chose three genes–the expression of which is very robustly repressed by EBNA3C –to explore the molecular interactions involved. Hitherto these genes had not been shown to require RBPJ for EBNA3C-mediated repression. We have described the sequence of events during repression and challenge a widely held assumption that if a protein interacts with RBPJ it would be recruited to DNA because of the intrinsic capacity of RBPJ to bind specific sequences. We show that interaction with RBPJ is essential for the repression of all three genes during the infection of B cells by EBV, but that RBPJ itself is only recruited to the genes when EBNA3C is functional. These data suggest an unexpectedly complex interaction of multiple proteins when EBNA3C prevents the expression of cellular genes.

## Introduction

Epstein-Barr virus (EBV) is a large DNA virus that belongs to the gamma subfamily of herpes viruses and infects persistently >90% of the human population. Infection with EBV is aetiologically associated with several types of human cancer, including Burkitt lymphoma, Hodgkin lymphoma, peripheral natural killer/T-cell lymphoma, nasopharyngeal and gastric carcinoma [1]. Infection of B cells with EBV results in activation and transformation of resting cells into proliferating B blasts, in which the viral genome resides as an extra-chromosomal episome within the nucleus. *In vivo*, early after infection, all EBV latency-associated genes are expressed, producing six EBV nuclear antigens [EBNA1, 2, 3A, 3B, 3C and leader protein (LP)], three latent membrane proteins (LMP1, 2A and 2B), two small non-coding RNAs (EBER1 and 2) and micro-RNA transcripts from the BamHI A region (BARTs) [1,2]. *In vitro*, infection of primary resting B cells with EBV creates continuously proliferating lymphoblastoid cell lines (LCL) that constitutively express all latency-associated EBV genes [1].

The genes encoding EBNA3A, 3B and 3C are arranged in tandem in the EBV genome and share the same gene structure with a short 5’ exon and a long 3’ exon. The proteins originate, through alternative splicing, from the B cell specific EBNA2/LP/3A/3B/3C transcription unit resulting in very long mRNAs initiated primarily from the Cp promoter. There are only a few copies of EBNA3 mRNAs in LCLs, probably due to tight transcriptional regulation–for example it has been reported that less than 3 mRNA copies of EBNA3C per cell can be detected [3]–and associated with slow turnover of the proteins [4]. The EBNA3s form a family of transcriptional co-regulators that can cooperate to regulate host gene expression [5–7]. EBNA3 proteins do not bind DNA directly, but are assumed to be tethered to target genes by associating with DNA sequence-binding factors, an example being RBPJ (also known as RBP-jk, CBF1, CSL, Suppressor of Hairless and Lag1) [8–12].

RBPJ is a component of the Notch signalling pathway that was first discovered in *Drosophila*, but is highly conserved across species and has an important role in developmental processes in embryonic and adult tissue, *e*.*g*. cell lineage decisions (reviewed in [13,14]). In vertebrates–in the absence of active Notch signalling–RBPJ represses Notch target genes through interaction with TFIIA and TFIID to prevent transcription [15] and also recruitment of repressor complexes containing histone deacetylase 1 and 2 (HDAC1 and 2), silencing mediator of retinoid and thyroid hormone receptors (SMRT/NcoR), SMRT/HDAC1-associated repressor protein (SHARP/MINT/SPEN), CBF1-interacting co-repressor (CIR), C-terminal binding protein (CtBP), CtBP-interacting protein (CtIP) and KyoT2 [16–20]. Ligand binding to the Notch receptors induces a series of proteolytic cleavages of the receptor resulting in the release of Notch intracellular domain (NICD) from the cell membrane [21–23]. NICD translocates into the nucleus where it binds to RBPJ via its RBPJ-associated molecule (RAM) domain WΦP (Φ = hydrophobic residue) motif (WFP) [24] and via its ankyrin repeats [25–27]. Binding of NICD to RBPJ disrupts the association with repressor complexes [16] and additional binding to the strong co-activator Mastermind [28–30] leads to formation of a stable activating complex and full activation of the repressed Notch signalling target genes.

EBNA2 also binds to RBPJ via a RAM domain WWP motif [31–34]. EBNA2, one of the first viral genes expressed after infection of B cells and a transcriptional transactivator of the other latent viral genes as well as cellular genes, operationally resembles NICD [35]. All the EBNA3s share a highly conserved N-terminal homology domain (HD) that contains RBPJ binding sites [9,11]. EBNA2 and EBNA3s, however, form mutually exclusive complexes through competitive binding to the same binding site on RBPJ [8,36]. EBNA3/RBPJ complexes were shown to disrupt DNA binding of RBPJ *in vitro*, in electrophoretic mobility shift assays [8,9,37] and to repress EBNA2-mediated activation in transient reporter assays [8,11,37,38]. This repression is dependent on the ability of EBNA3 to bind to RBPJ. Mutation of four core residues within the HD of EBNA3C from _209_TFGC to _209_AAAA (HDmut) that affect binding to RBPJ produced a protein that did not disrupt RBPJ/DNA binding and that failed to repress EBNA2-mediated transcriptional activation in transient reporter assays [11,39]. The HDmut EBNA3C also failed to sustain LCL proliferation when transfected into LCL with conditional EBNA3C after inactivation of EBNA3C [40,41]. In addition to the earlier identified core _209_TFGC motif [11], Calderwood and colleagues more recently identified a RAM-like motif (_227_WTP) in EBNA3C but not EBNA3A and EBNA3B [42]. The W227S mutant EBNA3C successfully repressed EBNA2-mediated transcriptional activation in transient reporter assays and sustained LCL proliferation in back-complementation assays, however, both _209_AAAA and W227S mutations were required for an effective loss of RBPJ binding as determined by co-immunoprecipitation [42].

Originally, a model was proposed in which EBNA2 acts as a viral analogue of NCID, producing transcriptional activation when bound to RBPJ; this is counteracted by competitive binding of EBNA3s to RBPJ and destabilisation of RBPJ binding to DNA [1,43,44]. An alternative model was then proposed in which EBNA3s directly recruit repressors to RBPJ that remains statically bound to its responsive elements [45]. When targeted directly to DNA by fusion with the DNA-binding domain of Gal4, all EBNA3 proteins exhibit strong repressor activity in reporter assays [37,46,47]. Moreover, EBNA3C can interact with cellular factors that are involved in transcriptional repression, these include HDAC1, HDAC2, CtBP, Sin3A and NcoR [48–50]–this would be consistent with the repressor recruitment model. However, it is fair to say that currently the role of RBPJ in gene regulation by EBNA3C remains largely unknown.

From previous microarray analyses in EBNA3A knockout (KO) LCL [5], BL31 infected with EBNA3A or EBNA3C KO viruses [6], BJAB cells stably expressing EBNA3C [51] and EBNA3C-conditional LCL [52] it is known that EBNA3A and EBNA3C repress *ADAM28* and *ADAMDEC1*, two members of a disintegrin and metalloprotease (ADAM) family that are encoded in adjacent genomic loci. McClellan and colleagues identified an intergenic EBNA3 binding site that loops to the transcription start site (TSS) of both genes only in the presence of EBNA3C and repression involved reduced levels of activation-associated H3K9/14ac mark and increased levels of the repressive H3K27me3 mark within *ADAM28* and at the TSS of *ADAMDEC1* [51,53]. However, these observations were obtained from stable transfectants of EBNA3C in the EBV-negative B cell lymphoma line BJAB in the absence of the other latent viral gene products and nothing is known about the temporal sequence of events at these regulatory sites and TSS, or the factors that are involved in EBNA3C-mediated gene repression early after infection of primary B cells with EBV. In addition to confirming repression of both ADAMs, a microarray study of EBNA3C-conditional LCL identified *COBLL1* as the gene most robustly repressed by EBNA3C (S1 Table, [52]). The function of the *COBLL1* gene product is unknown and it has not previously been characterised as an EBNA3C repressed gene. Based on the previous studies and the microarrays, we selected *ADAM28*, *ADAMDEC1* and *COBLL1* in order to explore in more detail the temporal sequence of events and factors that are involved in EBNA3C-mediated gene regulation.

Here, we show that all three genes are highly repressed in B cells following infection of primary CD19^+^ cells with EBV, only when EBNA3C is expressed and functional. Using LCLs conditional for EBNA3C function we could show for a first time that this system can be used efficiently to replicate EBNA3C-mediated changes in gene expression very similar to those seen early after infection of primary B cells with EBV. Using the conditional system we were able to explore further the temporal changes in epigenetic marks at regulatory elements and TSSs leading to repression of transcription and showed that it involved two-steps, rapid initial loss of activation-associated histone marks that led to repression of mRNA expression, followed by recruitment of polycomb proteins and increases of repressive histone H3K27me3 mark. Furthermore, we show that RBPJ is only recruited when EBNA3C is functional and that repression is absolutely dependent on the ability of EBNA3C to bind to RBPJ. This is the first time that EBNA3C-mediated transcriptional repression has been described in such detail and it provides novel insights into temporal sequence of events occurring early after infection and the dynamic role of RBPJ in EBV-mediated gene repression.

## Results

### EBNA3A and EBNA3C co-repress *ADAM28* and *ADAMDEC1*, but only EBNA3C is required to repress *COBLL1* in primary B cell infection

**Fig 1:**
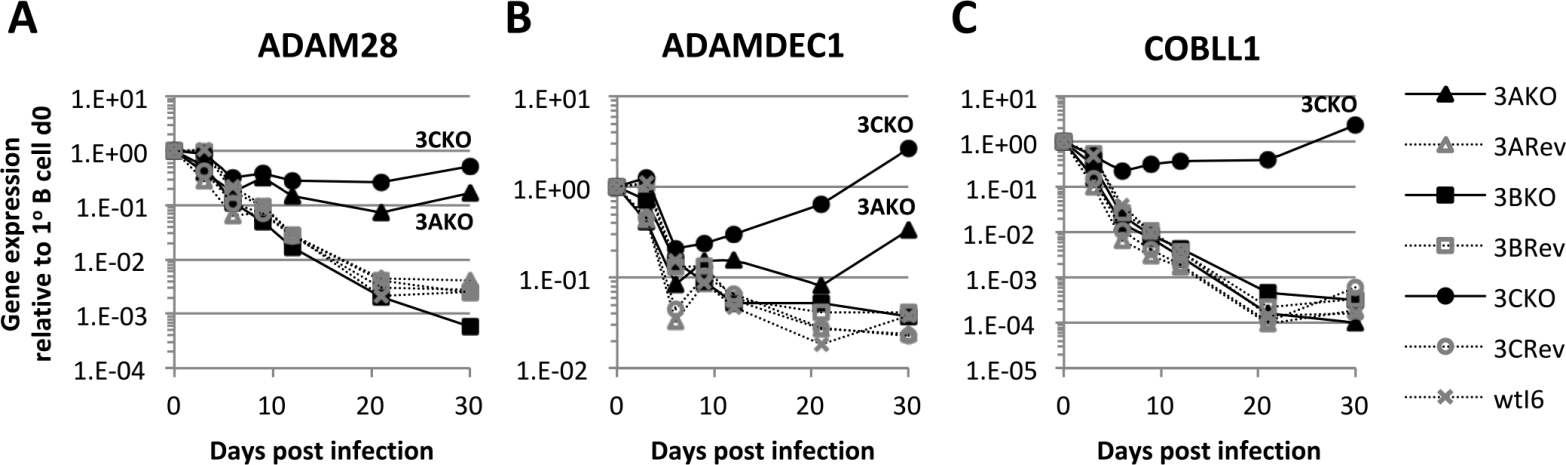
Repression of *ADAM28*, *ADAMDEC1* and *COBLL1* after infection of primary B cells with EBV. Infection of primary B cells with wild type (wtI6), recombinant EBNA3A, EBNA3B or EBNA3C knockout (KO) or revertant (Rev) recombinant viruses. Gene expression for *ADAM28* (**A**), *ADAMDEC1* (**B**) and *COBLL1* (**C**) was normalised to *GAPDH* and is shown relative to uninfected primary B cells. Normalisation to other internal control genes (*e*.*g*. *GNB2L1*) showed very similar results.

Interrogation of Affymetrix Exon 1.0 ST microarray analysis from the EBNA3C conditional system (3CHT) indicated that *COBLL1*, *ADAM28* and *ADAMDEC1* required expression of functional EBNA3C for very significant levels of repression in LCL (S1 Table; http://www.epstein-barrvirus.org.uk). In order to establish that *ADAM28*, *ADAMDEC1* and *COBLL1* are regulated by EBNA3C during viral infection of B cells–and to determine whether EBNA3A and/or EBNA3B are involved in the regulation–primary CD19^+^ cells were infected with previously characterised wild type (B95.8-BAC) EBV or recombinant EBNA3 KO or revertant (Rev) viruses (Fig 1A–1C) [54]. Infections with wild type, Rev and EBNA3B KO viruses resulted in a reduction of both ADAM28 (2–3 log fold) and ADAMDEC1 (1–2 log fold) levels of mRNA, over a period of 30 days after infection. In the absence of EBNA3C (3CKO) and to a lesser extent EBNA3A (3AKO) there was a failure to repress *ADAM28* (Fig 1A) and *ADAMDEC1* (Fig 1B)–this is consistent with reports derived from stable cell lines [51]. All infections with EBNA3C competent viruses led to a remarkable 3–4 log fold reduction of COBLL1 mRNA over the same period of time, but this was not seen after infection with 3CKO virus–here the levels detected in primary B cells were maintained (Fig 1C). These differences in gene expression between the various virus infections were not observed for the two control genes *ALAS1* (S1A Fig) and *GNB2L1* (S1B Fig), neither of which are known to be targets of EBNA3 proteins or EBV.

### LCLs with conditional EBNA3C recapitulate repression of *ADAM28*, *ADAMDEC1* and *COBLL1* seen in primary B cell infection

**Fig 2:**
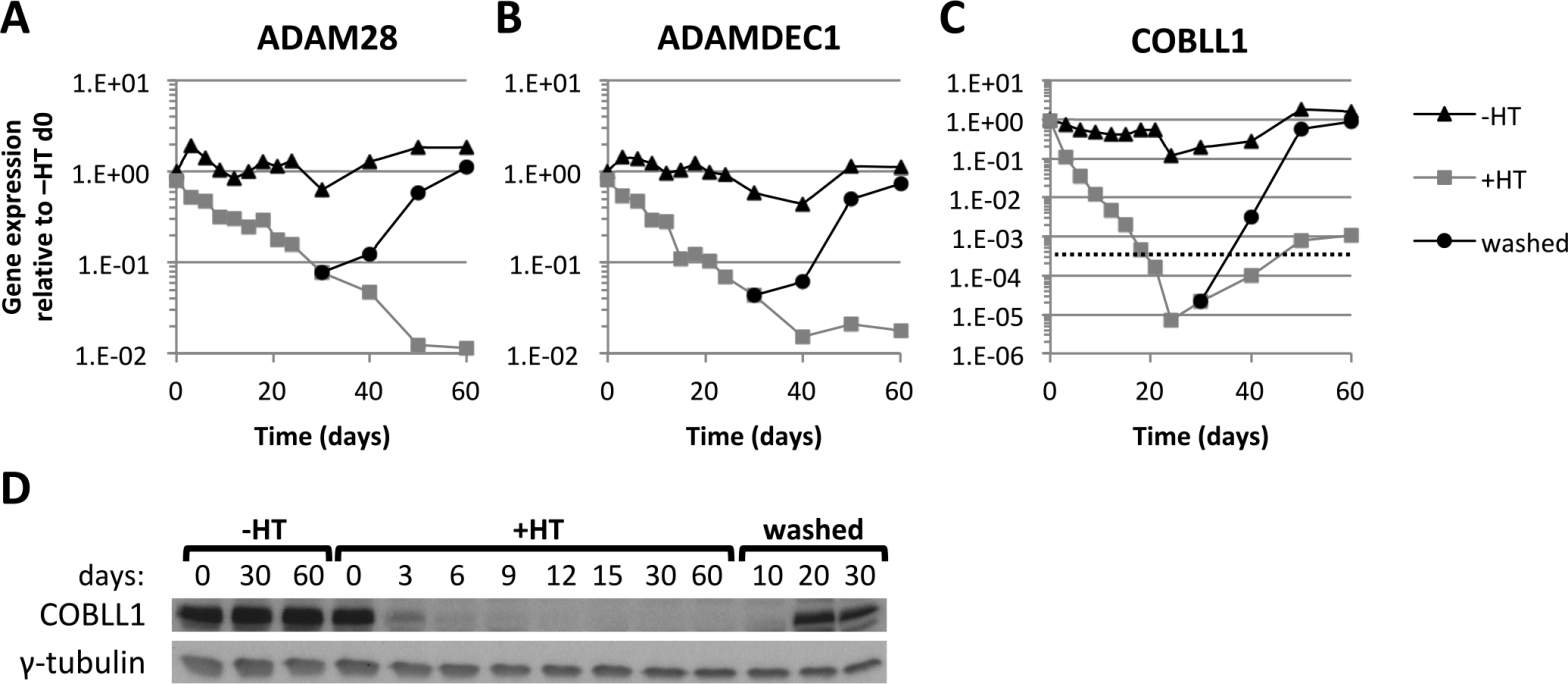
Repression of *ADAM28*, *ADAMDEC1* and *COBLL1* in EBNA3C conditional cell line. (**A-C**) Time-course using EBNA3C-conditional LCL 3CHT A13. Cells were grown over 60 days either in absence of HT (-HT), presence of HT (+HT) or with HT removed after 30 days +HT (washed). Gene expression for *ADAM28* (**A**), *ADAMDEC1* (**B**) and *COBLL1* (**C**) was normalised to *GAPDH* and is shown relative to -HT on day 0. The dashed line indicates gene expression levels of the repressed state, which equals the limit of detection for *COBLL1* –values below this line become extremely unreliable. (**D**) COBLL1 and γ-tubulin protein expression during 3CHT A13 time-course in the absence of HT (-HT), presence of HT (+HT) or HT washed away on day 30 (washed).

After establishing that all three genes were robustly repressed after infection of primary B cells with EBV, which confirmed previous findings from stable cell lines that *ADAM28* and *ADAMDEC1* were repressed by EBNA3C and EBNA3A and identifying that *COBLL1* was repressed by EBNA3C alone, we wanted to determine whether it was possible to recapitulate this repression in LCLs carrying EBV-recombinants conditional for EBNA3C (3CHT). In this cell line, EBNA3C activity is conditional on the presence of 4-hydroxytamoxifen (HT) and proliferation of the cells does not decrease in its absence due to the homozygous deletion of p16^INK4A^, a primary target of EBNA3C [52]. This cell line could therefore be established having never expressed functional EBNA3C (3CHT A13) and the expression of *ADAM28*, *ADAMDEC1* and *COBLL1* in this cell line is similar to uninfected primary B cells. Activation of EBNA3C by the addition of HT to these cells (+HT) resulted in a 2-log fold repression of *ADAM28* (Fig 2A), *ADAMDEC1* (Fig 2B) and a 4-log fold repression of *COBLL1* (Fig 2C), which was not seen when EBNA3C was kept inactive (-HT) over this 60-day period. The repression of all three genes was fully reversible. Inactivation of EBNA3C, by washing out HT on day 30, led to an increase in expression of mRNAs corresponding to *ADAM28*, *ADAMDEC1* and *COBLL1* up to levels similar to those at the start of the time-course. The observed repression of *ADAM28*, *ADAMDEC1* and *COBLL1* in the 3CHT system was a direct consequence of the HT-induced activation of EBNA3C, because adding HT to a non-conditional EBNA3C KO LCL, grown out from the primary B cell infection (see below), did not change the expression levels of any of the three genes (S1C–S1E Fig).

The repression of *COBLL1* by EBNA3C could also be observed at the protein level. Over the 3CHT A13 60-day time-course, COBLL1 protein levels quickly disappeared. They were barely detectable three days after activation of EBNA3C (by the addition of HT) and were completely undetectable thereafter. However, COBLL1 protein did not reappear until about 20 days after inactivation of EBNA3C (Fig 2D). In addition, a rare LCL that grew from an infection of primary B cells with EBNA3C KO virus showed that only EBNA3C-deficient LCLs expressed COBLL1 (S2A Fig). Consistent with previous published data [52], cells from the EBNA3C KO virus infection underwent the expected crisis around 3–4 weeks post-infection but in a single experiment a sub-population survived and grew into a stable LCL. Immunoblot analysis revealed–in addition to high levels of COBLL1 –low levels of retinoblastoma protein (Rb), phosphorylated Rb (P-Rb) and loss of p16^INK4a^ in this unusual LCL (S2A Fig)–we and others have seen this type of clonal selection, more frequently, with cells infected with EBNA3A KO virus [5,55]. It should be noted that protein expression data for ADAM28 and ADAMDEC1 was not included because, in our hands, the commercial antibodies that we tested did not produce convincing or reproducible results.

The results obtained from the 3CHT A13 time-course were highly reproducible, not only in the same cell line, but also in 3CHT C19, another 3CHT cell line created by an independent 3CHT recombinant virus clone on the p16-null background, but grown out in the presence of HT and then washed (S1F–S1H Fig). The dynamic range of *COBLL1* repression was remarkable, following a similar highly exponential repression profile in both 3CHT A13 and C19 conditional cell lines and also in newly infected primary B cells (S3A Fig). Repression of *ADAM28* and *ADAMDEC1* was rather more variable between cell lines, but showed a similar exponential repression profile (S3B and S3C Fig). Taken together these results showed for the first time, that the EBNA3C conditional cell lines could be used to recapitulate efficiently EBNA3C-mediated gene repression observed in B cells early after infection with EBV.

### EBNA3C is targeted to regulatory sites, recruits polycomb proteins and induces changes in epigenetic histone marks

**Fig 3:**
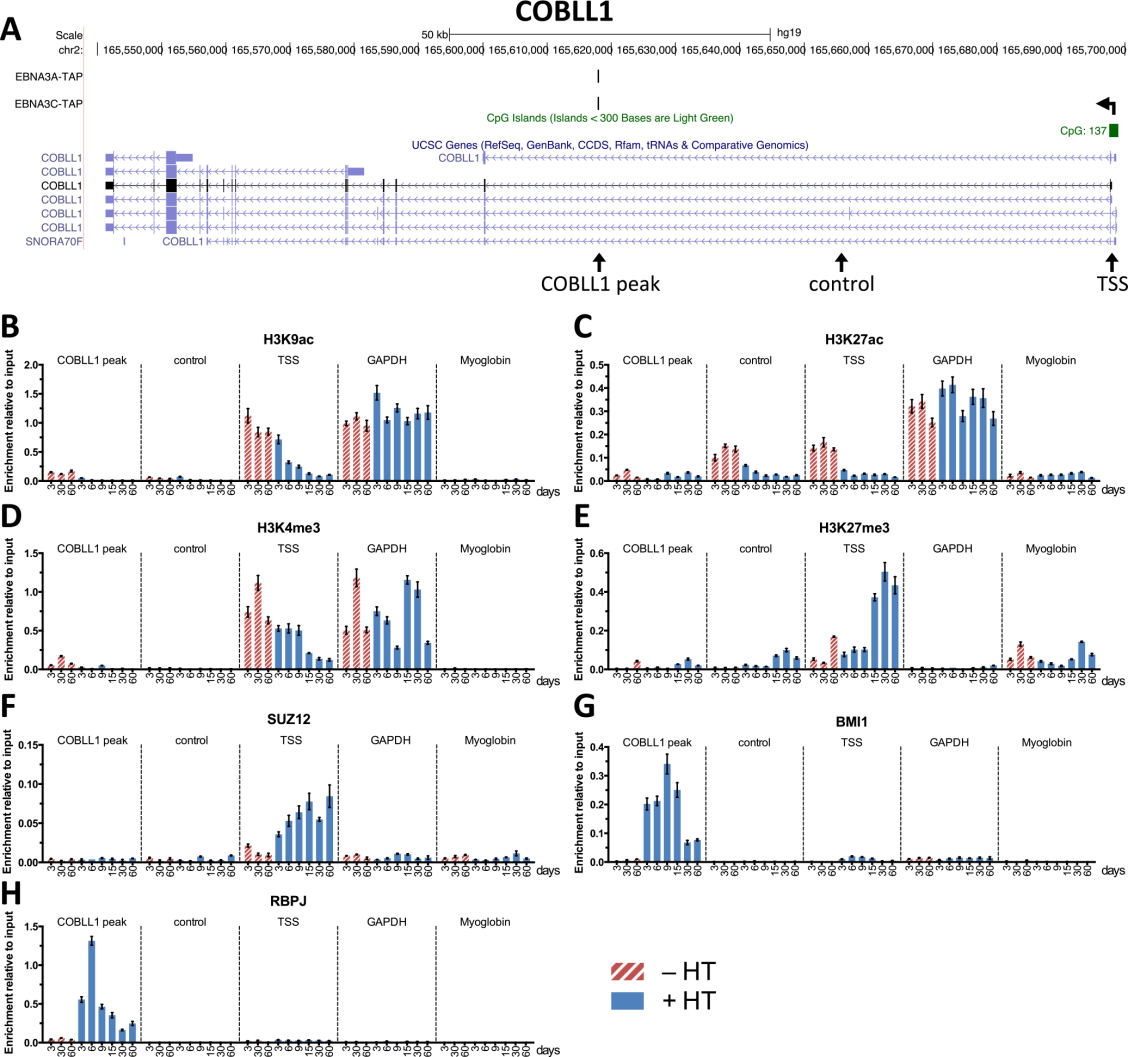
Epigenetic changes and factor accumulation at sites within the *COBLL1* locus during the 3CHT A13 time-course. (**A**) UCSC genome browser overview of *COBLL1* genomic locus showing ChIP-seq tracks for EBNA3A-TAP, EBNA3C-TAP, the TSS (horizontal arrow) and CpG islands. Primer locations for ChIP-qPCR (*COBLL1* peak, control and TSS) are indicated (vertical arrows below UCSC genes track). (**B-H**) ChIP for H3K9ac (**B**), H3K27ac (**C**), H3K4me3 (**D**), H3K27me3 (**E**), SUZ12 (**F**), BMI1 (**G**) and RBPJ (**H**) on samples from 3CHT A13 time-course at locations within the *COBLL1* locus, at *GAPDH* or *myoglobin* as indicated. Cells were grown in the absence (-HT) or presence of HT (+HT) and numbers indicate the day of harvest. ChIP values represent enrichment relative to input ± standard deviations of triplicate qPCR reactions for ChIP and input of each sample.

Having shown that the EBNA3C conditional system could be efficiently used to replicate gene expression changes seen early after EBV infection of primary B cells, we wanted to determine the sequence of events that led to such robust repression of *COBLL1* and the *ADAM28*-*ADAMDEC1* locus by employing chromatin immunoprecipitations (ChIP) on samples harvested throughout the 3CHT A13 time-course. *COBLL1* is located on chromosome 2, has multiple transcript variants and a CpG island around the promoter region. ChIP coupled to high throughput DNA sequencing (ChIP-seq) using LCLs expressing tandem-affinity purification (TAP) tagged EBNA3s ([56] and K. Paschos *et al*., manuscript in preparation) identified a single intragenic EBNA3A and EBNA3C binding site, hereafter called the *COBLL1* peak (Fig 3A). This binding site was confirmed in the LCLs used here by ChIP-qPCR (S4 Fig). ChIP analysis on samples from the 3CHT A13 time-course and probed across the *COBLL1* locus, showed that after activation of conditional EBNA3C, there was a sustained decrease in the activation-associated histone marks H3K9ac, H3K27ac and H3K4me3 and an increase in the repressive histone mark H3K27me3 primarily at the TSS of *COBLL1* (Fig 3B–3E). These changes were not observed at *GAPDH* or *Myoglobin*, two controls for expressed and repressed genes, respectively. Next, since H3K27me3 is catalysed by polycomb repressive complex 2 (PRC2) and polycomb complexes were previously found to be involved in the repression of *BCL2L11* (BIM) [57] and *CDKN2A* (p16^INK4A^) [55] we explored whether they also play a role in the repression of *COBLL1*. ChIP for PRC2-family member SUZ12 showed increased enrichment at the TSS of *COBLL1* as it is repressed (Fig 3F). In contrast and rather unexpected, ChIP for the polycomb repressive complex 1 (PRC1) family member BMI1 revealed recruitment of BMI1 to the *COBLL1* peak, but not the TSS, with highest BMI1 levels nine days after EBNA3C activation (Fig 3G). Finally, since the EBNA3 proteins cannot bind directly to DNA and RBPJ is the most well characterised DNA binding factor to which they have all been reported to bind, ChIP for RBPJ was performed. This revealed that RBPJ accumulated on the *COBLL1* peak, but only when EBNA3C was functional–with the highest levels appearing six days after activation of EBNA3C by HT (Fig 3H).

**Fig 4:**
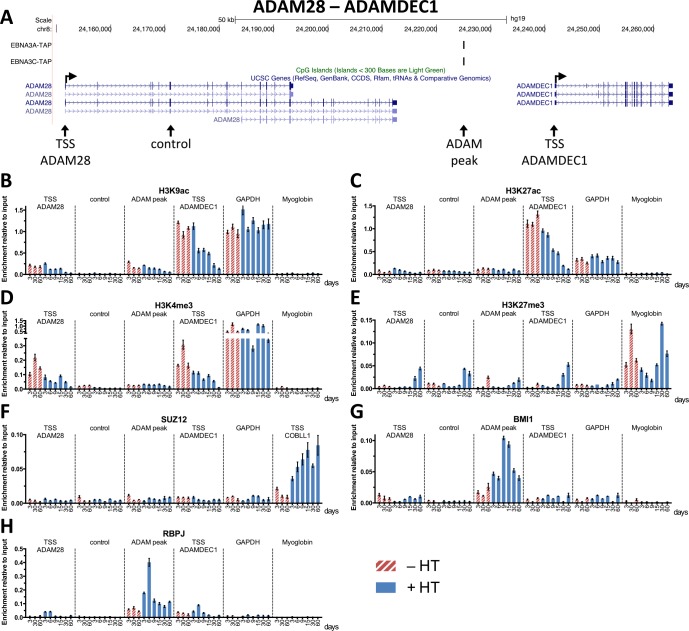
Epigenetic changes and factor accumulation at sites within the *ADAM28-ADAMDEC1* locus during the 3CHT A13 time-course. (**A**) UCSC genome browser overview of *ADAM28-ADAMDEC1* genomic locus showing ChIP-seq tracks for EBNA3A-TAP, EBNA3C-TAP, the TSSs (horizontal arrows) and CpG islands. Primer locations for ChIP-qPCR (TSS *ADAM28*, control, *ADAM* peak and TSS *ADAMDEC1*) are indicated (vertical arrows below UCSC genes track). (**B-H**) ChIP for H3K9ac (**B**), H3K27ac (**C**), H3K4me3 (**D**), H3K27me3 (**E**), SUZ12 (**F**), BMI1 (**G**) and RBPJ (**H**) on samples from 3CHT A13 time-course at locations across the *ADAM28-ADAMDEC1* locus, at *GAPDH* or *myoglobin* as indicated. Cells were grown in the absence (-HT) or presence of HT (+HT) and numbers indicate the day of harvest. ChIP values represent enrichment relative to input ± standard deviations of triplicate qPCR reactions for ChIP and input of each sample.

*ADAM28* and *ADAMDEC1* are encoded on chromosome 8 and also have multiple transcript variants, but no distinct CpG island. Previous studies by McClellan and colleagues [51] identified an intergenic EBNA3A and EBNA3C binding site (hereafter called the *ADAM* peak), which was also detected in our ChIP-seq performed on EBNA3A-TAP and EBNA3C-TAP LCLs (Fig 4A) and confirmed by ChIP-qPCR (S4 Fig). ChIP for activation associated histone marks on samples of the 3CHT A13 time-course and across the *ADAM* locus again showed a loss of H3K9ac, H3K27ac and H3K4me3 largely at the TSS of *ADAMDEC1*, but also *ADAM28*, when EBNA3C was made functional by the addition of HT (Fig 4B–4D). There was an increase in the repressive H3K27me3 mark across the *ADAM28-ADAMDEC1* locus, but at considerably lower levels than seen at the *COBLL1* TSS (Fig 4E). This is consistent with previous data from McClellan and colleagues that showed less H3K9/14ac and increased H3K27me3 in a stable EBNA3C expressing BJAB cell line [51]. Unlike at the *COBLL1* locus, no increase in SUZ12 enrichment could be detected across the *ADAM28-ADAMDEC1* locus (Fig 4F). However, similar to *COBLL1*, ChIP for BMI1 revealed recruitment to the *ADAM* peak (but again not to either TSS) with highest levels nine days after EBNA3C activation (Fig 4G). Although repression of this locus had not been previously described as being RBPJ-dependent, as with *COBLL1*, RBPJ enrichment also increased at the *ADAM* peak only when functional EBNA3C was induced, with highest levels appearing six days after activation (Fig 4H).

In order to confirm these temporal changes of histone marks and factor recruitment at both the *COBLL1* and *ADAM28-ADAMDEC1* loci, ChIP samples taken during a biological replicate time-course–using the 3CHT C19 LCL–were analysed in a similar way to the 3CHT A13 samples and showed very similar results (S5 and S6 Figs). Unfortunately, we were unable to reproducibly perform ChIP for EBNA3C using a commercial polyclonal antibody against EBNA3C that also precipitates EBNA3A and EBNA3B [53]. In order to reliably ChIP for EBNA3C during an extended time-course experiment, the conditional EBNA3C would also need to be TAP-tagged, but these viruses are not currently available.

Immunoblot analysis showed that there were no consistent changes to the levels of EBNA3A, EBNA3B, BMI1, SUZ12 and RBPJ proteins during either the 3CHT A13 or 3CHT C19 time-courses (S2B and S2C Fig).

### Loss of activation-associated histone marks correlates with repression of mRNA expression and precedes establishment of repressive histone marks

**Fig 5:**
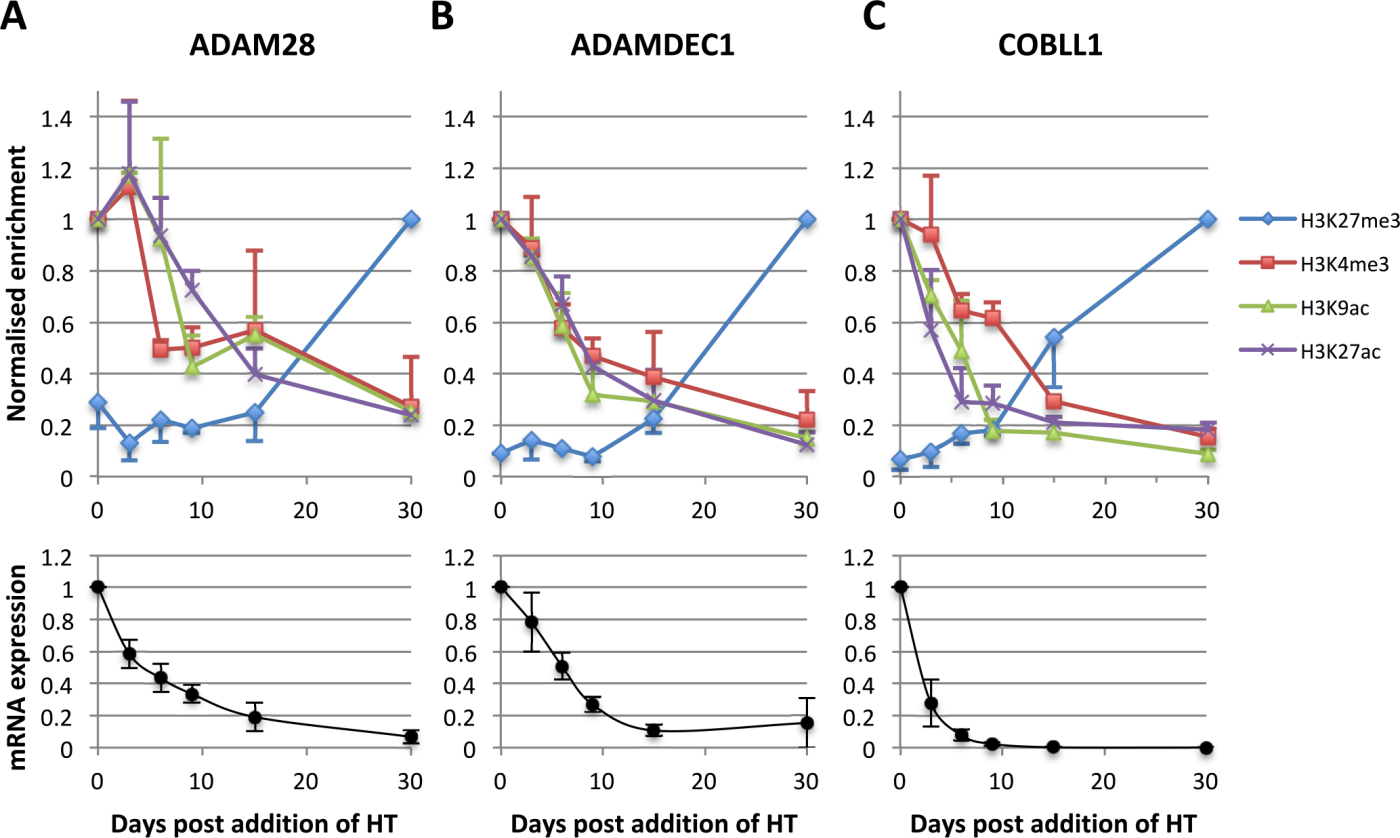
Correlation between changes of epigenetic marks over time at the TSS of *ADAM28*, *ADAMDEC1* and *COBLL1* with gene expression. ChIP-qPCR values (top) at the TSS of *ADAM28* (**A**), *ADAMDEC1* (**B**) and *COBLL1* (**C**) from 3CHT A13 and 3CHT C19 time-courses were normalised to day 30 +HT for the repressive histone mark H3K27me3 and to day three -HT for the activation-associated histone marks H3K4me3, H3K9ac and H3K27ac, which were then set as day 0. Mean values ± standard deviation from both replicate time-courses are shown. For better visual clarity, error bars are colour-matching the corresponding histone mark and only upper bars are shown for activation-associated marks and lower bars for H3K27me3. mRNA expression data for each gene (bottom) are shown as mean values ± standard deviation from three replicate time courses (3CHT A13 rep 1+2 and 3CHT C19).

The similarity of the two time-course experiments allowed a more detailed analysis of the temporal development of histone marks and factor recruitment to regulatory elements. For this, ChIP enrichment levels relative to input from both 3CHT A13 and 3CHT C19 time-courses were normalised. Activation-associated histone marks were expressed relative to the first time point–HT (day 3) and repressive histone marks relative to the last time point +HT (day 30). This revealed that at the TSS of all three genes loss of the activation-associated histone marks H3K9ac, H3K27ac and H3K4me3 preceded any increase in the repressive histone mark H3K27me3 (Fig 5A–5C top). Furthermore, comparison between the changes in histone marks and corresponding mRNA expression levels (Fig 5A–5C bottom) of each gene over time revealed that the initial repression of mRNA expression was caused by loss of all three activation-associated histone modifications (H3K9ac, H3K27ac and H3K4me3) and was independent of the appearance of the repressive H3K27me3 modification, which was only deposited after most mRNA was depleted.

**Fig 6:**
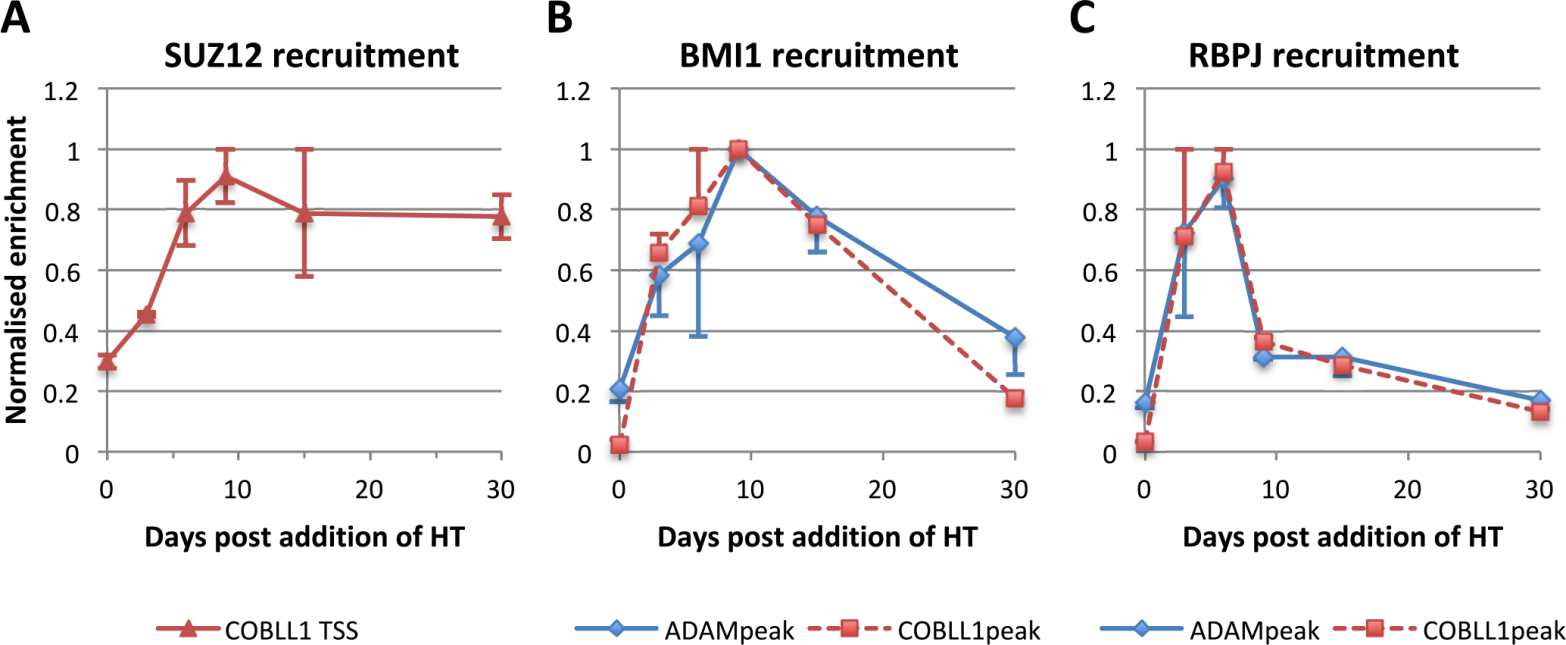
Recruitment of SUZ12, BMI1 and RBPJ to EBNA3C-binding peaks in *ADAM28-ADAMDEC1* and *COBLL1* loci. ChIP-qPCR values for SUZ12 at the TSS of *COBLL1* (**A**), BMI1 (**B**) and RBPJ (**C**) at the EBNA3C-binding peaks at *ADAM28-ADAMDEC1* (*ADAM* peak) and *COBLL1* (*COBLL1* peak) from 3CHT A13 and 3CHT C19 time-courses were normalised by setting the maximal ChIP enrichment level during each time course to one and by using -HT day three as representative value for day 0. For BMI1, day nine of the C19 time course was treated as an outlier and not taken into account. Mean values ± standard deviation from both replicate time-courses are shown. For better visual clarity, error bars are colour-matched to either *ADAM* peak (only lower bars displayed) or *COBLL1* peak (only upper bars displayed).

At the TSS of *COBLL1*, maximal SUZ12 enrichment levels were reached by day nine, which precedes the substantial increase in H3K27me3 at this site about 15 days after activation of EBNA3C (Fig 6A). Analysis of the BMI1 recruitment to both *ADAM* peak and *COBLL1* peak showed that maximal BMI1 levels were reached nine days after activation of EBNA3C (Fig 6B). However, these high BMI1 levels were not maintained and relative enrichment levels of BMI1 subsequently dropped, but they remained significantly higher than in cells where EBNA3C was kept inactive. Interestingly, comparing the recruitment profile of SUZ12 to the TSS of *COBLL1* with that of BMI1 to the *COBLL1* peak, it appeared that both increased simultaneously at the distinct sites, but in contrast to BMI1, SUZ12 levels were maintained at a high level for at least 60 days (Fig 3F). Analysis of the accumulation of RBPJ at both the *ADAM* and *COBLL1* peaks revealed again what appeared to be a transient recruitment of RBPJ with maximal enrichment 3–6 days after the addition of HT (Fig 6C). This preceded the recruitment of BMI1 to the two EBNA3-binding sites.

Taken together, these analyses showed consistently that activation-associated histone marks were removed first, consistent with this being the main cause for repression of mRNA expression, before repressive marks were established. Furthermore, the dynamic recruitment of both RBPJ and BMI1 was dependent on functional EBNA3C, with RBPJ recruitment probably preceding BMI1 and EBNA3C involved in recruiting both.

### Transient reporter assays recapitulate repression of *COBLL1* and *ADAM28* and show dependency on RBPJ

**Fig 7:**
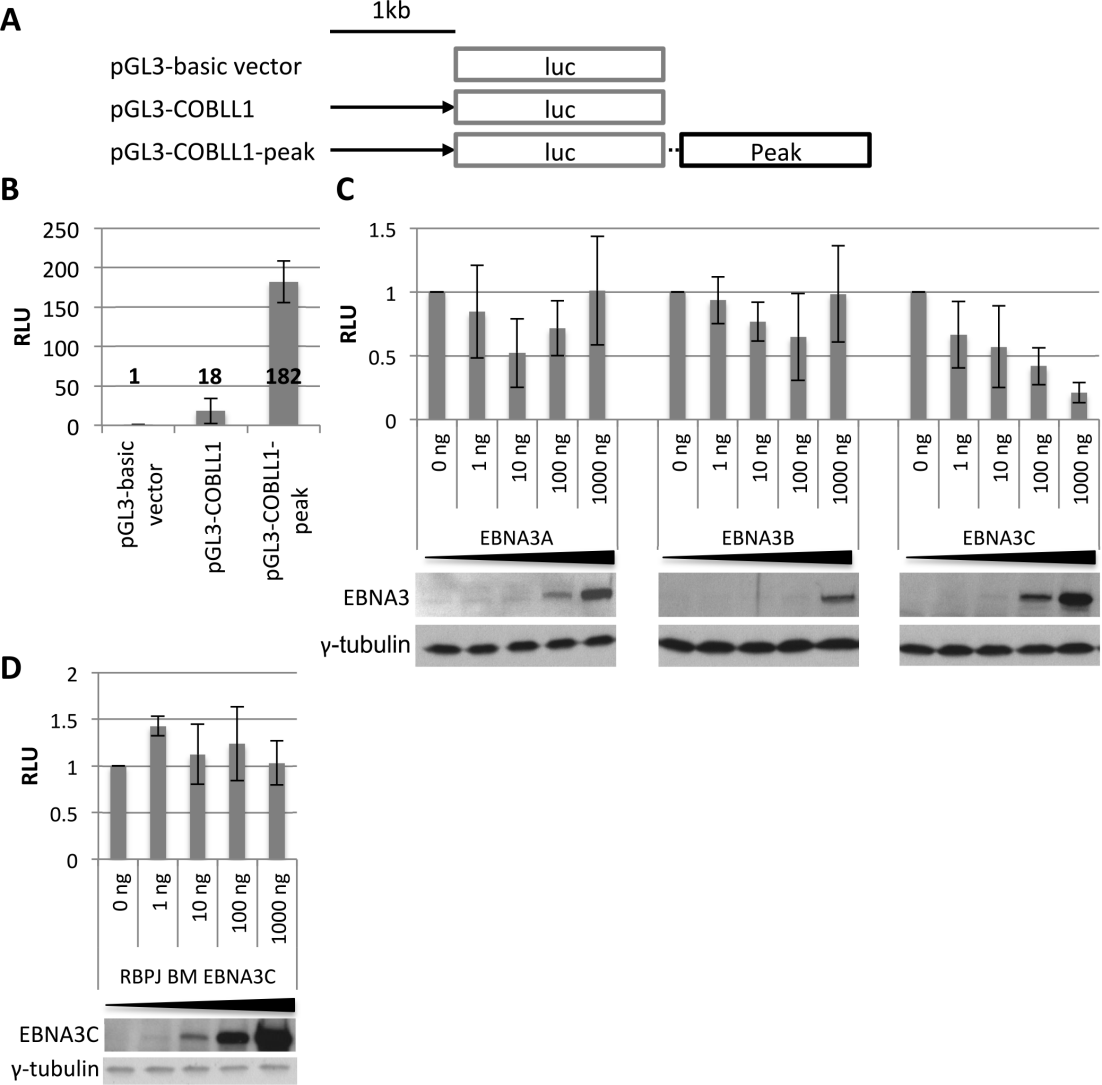
Repression of *COBLL1* transient reporter construct by EBNA3C is dependent on the *COBLL1* peak and RBPJ. (**A**) Schematic of luciferase vectors used in transient reporter assays. Black arrow represents 1 kb promoter region upstream of *COBLL1* TSS and black box 1.5 kb around EBNA3C-binding peak at *COBLL1* cloned into pGL3-basic vector. (**B**) Luciferase reporter assay in Raji using 1 μg of luciferase vector as indicated and expressed relative to pGL3-basic vector (n = 3). (**C**) Luciferase reporter assay in Raji using 1 μg of pGL3-*COBLL1*-peak luciferase vector and co-transfecting increasing amounts of EBNA3 expression plasmids as indicated (n = 4). (**D**) Luciferase reporter assay as in C but co-transfecting increasing amounts of RBPJ binding mutant (BM) of EBNA3C (n = 3). Immunoblots show corresponding EBNA3 protein and γ-tubulin as loading control. All luciferase units were normalised to beta-galactosidase units of the same transfection. Results are shown as mean ± standard deviation.

We were interested to see whether it was possible to recapitulate the repression of *COBLL1* and *ADAM28* in transient reporter assays. Therefore, initially, the promoter region of *COBLL1* was cloned upstream of a luciferase cassette either in the presence or absence of the *COBLL1* peak inserted downstream of luciferase (Fig 7A). The ideal B cell line for transient reporter assays is the readily transfectable EBV-negative Burkitt’s lymphoma cell line DG75 [58]. However, luciferase activity of the *COBLL1* construct was very low in this cell line, which made it impossible to study repression, perhaps because DG75 cells do not express endogenous *COBLL1*. Therefore, a panel of transfectable B cell lines was screened for luciferase activity of the *COBLL1* construct and it was found to be robust in the EBV-positive, but EBNA3C-null cell line Raji [59]. This expresses endogenous *COBLL1*. The presence of *COBLL1* peak in the plasmid led to an increase in luciferase activity (~10 fold) relative to the construct that only has the promoter region of *COBLL1* –indicating that, in the absence of EBNA3C, the *COBLL1* peak acts as an enhancer (Fig 7B). Co-transfection of expression plasmids for the EBNA3s along with the luciferase constructs that contain the *COBLL1* peak showed that EBNA3C repressed luciferase activity, but neither EBNA3A nor EBNA3B had this effect (Fig 7C). This was consistent with the results from the primary B cell infections presented above, confirming EBNA3C as sole repressor of *COBLL1*. Co-transfection of the *COBLL1* reporter with an expression plasmid encoding a mutant EBNA3C that is unable to bind to RBPJ based on the double mutant described in Calderwood *et al*. (see Introduction and Materials and Methods), failed to repress luciferase activity (Fig 7D).

**Fig 8:**
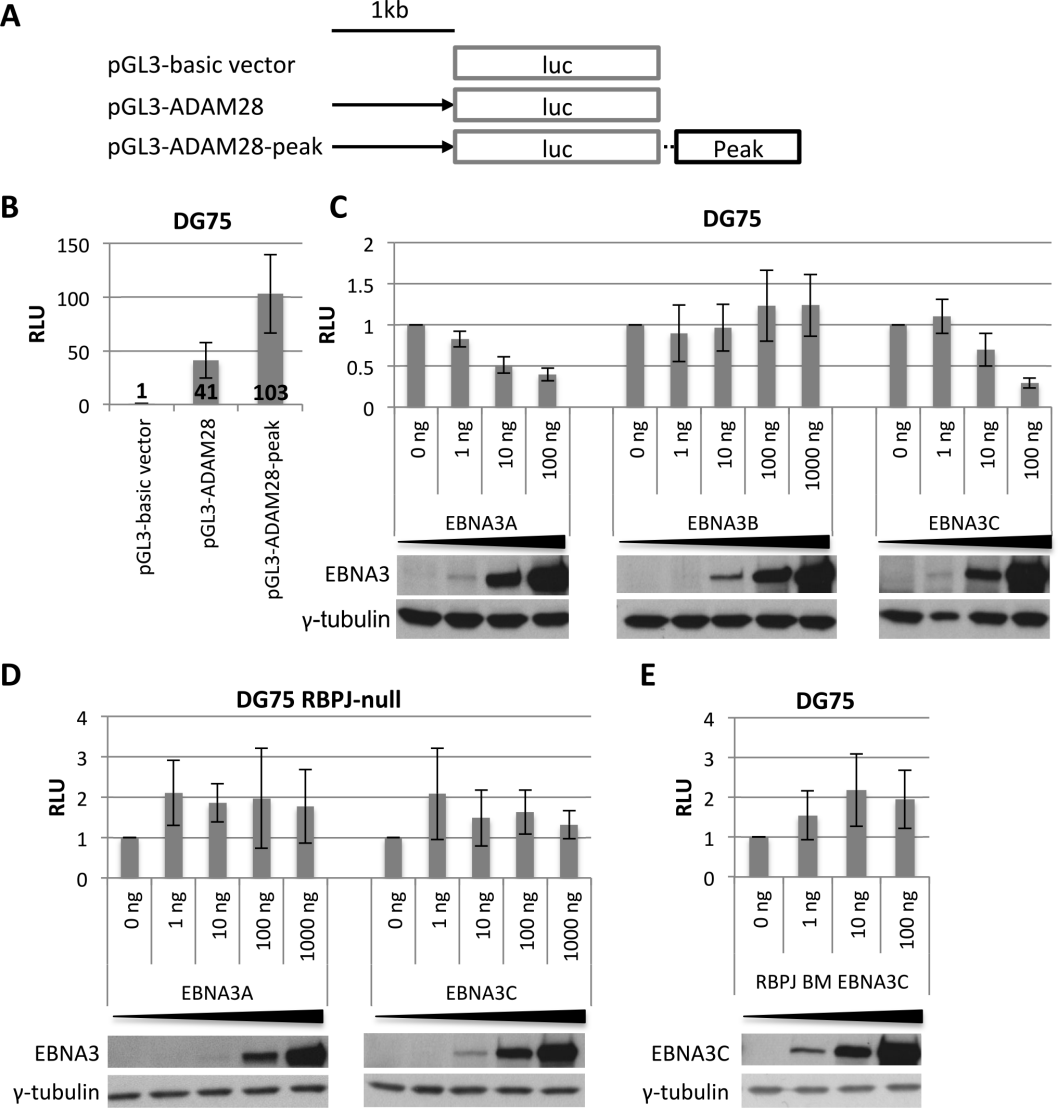
Repression of ADAM28 transient reporter construct by EBNA3A and EBNA3C is dependent on the *ADAM* peak and RBPJ. (**A**) Schematic of luciferase vectors used in transient reporter assays. Black arrow represents 1 kb promoter region upstream of *ADAM28* TSS and black box 1 kb around EBNA3C-binding peak at *ADAM28-ADAMDEC1* locus cloned into pGL3-basic vector. (**B**) Luciferase reporter assay in DG75 using 1 μg of luciferase vector as indicated and expressed relative to pGL3-basic vector (n = 6). (**C**) Luciferase reporter assay in DG75 using 1 μg of pGL3-*ADAM28*-peak luciferase vector and co-transfecting increasing amounts of EBNA3 expression plasmids as indicated (n = 3 for EBNA3A and EBNA3B, n = 6 for EBNA3C). (**D**) Luciferase reporter assays as in C for EBNA3A (n = 3) and EBNA3C (n = 4) but in RBPJ-null DG75. (**E**) Luciferase reporter assay as in C but co-transfecting increasing amounts of expression plasmid encoding for RBPJ binding mutant (BM) of EBNA3C (n = 3). Immunoblots show corresponding EBNA3 protein and γ-tubulin as loading control. All luciferase units were normalised to beta-galactosidase units of the same transfection. Results are shown as mean ± standard deviation.

In order to recapitulate the repression of ADAM28 a similar approach was used in the EBV-negative Burkitt’s lymphoma cell line DG75 [58], here luciferase activity of the *ADAM28* construct was robust (Fig 8A). Again the presence of the *ADAM* peak included downstream of the luciferase gene led to an increase in luciferase activity, but this was not as substantial as for COBLL1 (<3 fold) (Fig 8B). For this construct, co-transfection of plasmids expressing either EBNA3A or EBNA3C, but not those expressing EBNA3B, resulted in a reduction in luciferase activity (Fig 8C)–again consistent with results from the primary B cell infection experiments. As for the *COBLL1* reporters, the repression of the *ADAM* reporter was dependent on the presence of RBPJ, since neither EBNA3A nor EBNA3C induced a reduction in luciferase activity in RBPJ-null DG75 cells (SM224.9 [60], Figs 8D and S2D). Moreover, co-transfection of the *ADAM* reporter with the plasmid expressing the EBNA3C RBPJ-binding mutant also failed to repress luciferase activity (Fig 8E).

These results showed that transient reporter assays can be used to recapitulate the repression of *COBLL1* by EBNA3C and the repression of *ADAM28* by both EBNA3A and EBNA3C and that the ability of EBNA3s to bind and recruit RBPJ was likely to be important for the repression of both loci in the context of latent EBV infection.

### Recombinant EBV with RBPJ binding mutant of EBNA3C fails to repress *ADAM28*, *ADAMDEC1* and *COBLL1*

**Fig 9:**
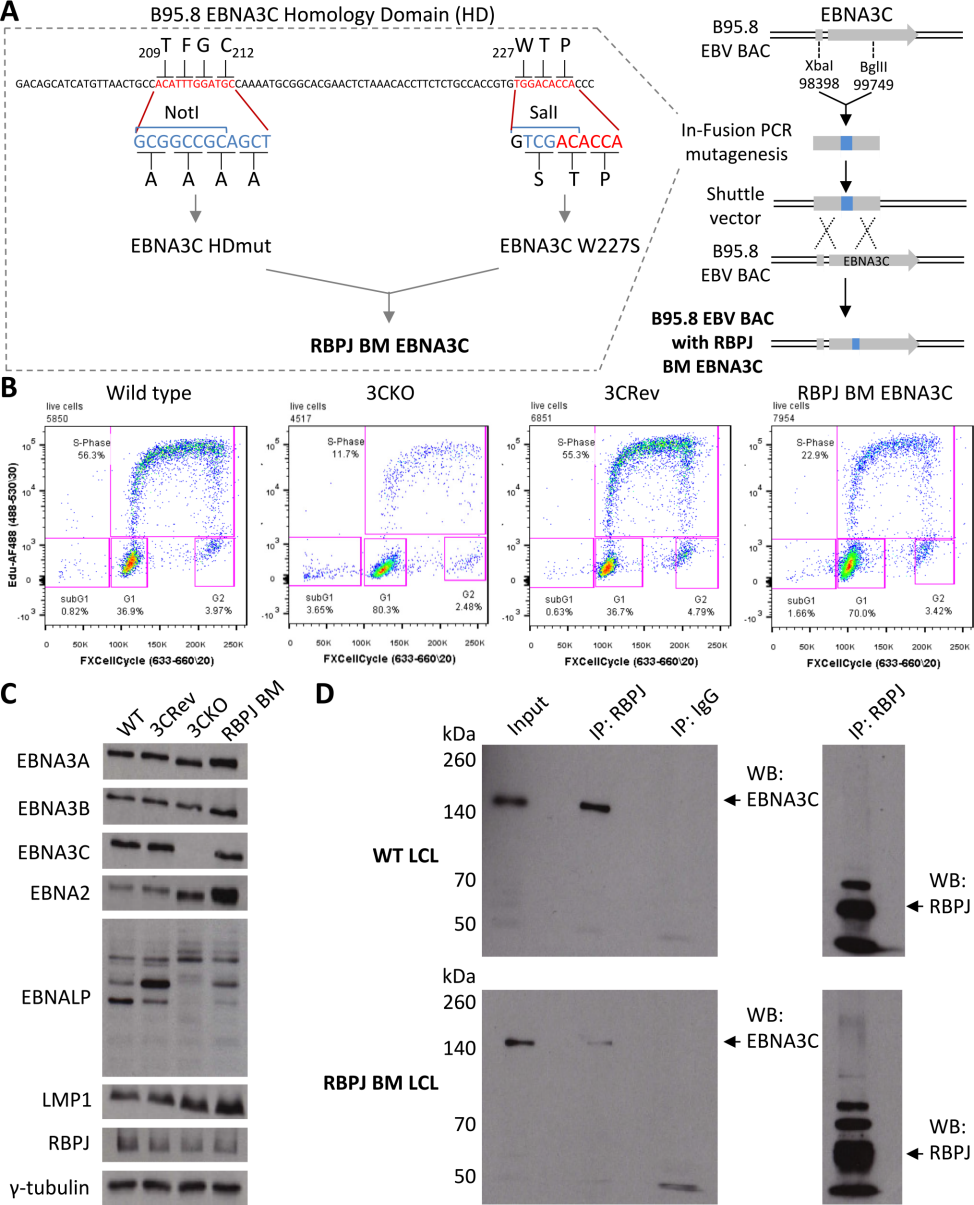
Construction of recombinant EBV with RBPJ binding mutant EBNA3C and validation of established LCL. (**A**) Schematic representation of the construction of the EBV recombinant encoding the EBNA3C binding mutant (EBNA3C BM) incapable of binding RBPJ. The N-terminal XbaI/BglII fragment of EBNA3C was excised from the B95.8 EBV bacterial artificial chromosome (BAC) and used as template for In-Fusion based mutagenesis. The two RBPJ binding sites, _209_TFGC and _227_WTP, of EBNA3C were mutated to _209_AAAA and W227S based on previous studies [11,42]. The nucleotide sequence of the wild type EBV BAC is shown in red and the mutated sequence in blue. Mutations were introduced in a two-step In-Fusion based mutagenesis process, with the _209_AAAA mutation (homology domain (HD) mutant) generated first, introducing a NotI restriction site, followed by the W227S mutation, introducing a SalI restriction site. The resulting RBPJ binding mutant EBNA3C was reintroduced into the B95.8 EBV BAC by RecA mediated homologous recombination. (**B**) Cell proliferation assay at day 36 after infection of primary B cells with wild type, EBNA3C knockout (3CKO), EBNA3C revertant (3CRev) or RBPJ binding mutant EBNA3C (RBPJ BM) recombinant viruses. Live cells were analysed for proliferation by measuring EdU incorporation and DNA content by FxCycle Far red DNA stain. Gates show populations of cells in sub-G1, G1, S-phase or G2/M phase. (**C**) Immunoblot for EBNA3A, EBNA3B, EBNA3C, EBNA2, EBNALP, LMP1, RBPJ and γ-tubulin on LCLs established from primary B cell infection with wild-type (WT), EBNA3C Revertant (3CRev), EBNA3C knockout (3CKO) and RBPJ binding mutant EBNA3C (RBPJ BM) EBV. (**D**) Immunoprecipitation (IP) of RBPJ or antibody isotype control (IgG) in WT LCL or RBPJ BM LCL and western blotting (WB) for EBNA3C or RBPJ as indicated. Input represents 10% of lysate used in IPs and arrows indicate bands of EBNA3C or RBPJ.

In order to determine whether binding of EBNA3C to RBPJ is necessary for the repression of the endogenous *COBLL1* and *ADAM28-ADAMDEC1* locus in the context of infection, a new EBV recombinant encoding the RBPJ binding mutant of EBNA3C (RBPJ BM EBNA3C) was constructed. The RBPJ BM EBNA3C was based on the double mutant described by Calderwood and colleagues [42] and comprised the newly identified W227S mutation and the previously identified mutation of residues _209_TFGC→AAAA ([11], see Introduction and Fig 9A). NotI and SalI restriction sites were introduced in order to allow restriction digest confirmation of successful mutagenesis (S7A Fig) and rescued BACs from HEK293 virus producing clones (S7B Fig) that maintained general BAC integrity. DNA sequencing of rescued episomes confirmed the mutations that had been engineered.

Infection of primary CD19^+^ cells with this RBPJ BM EBNA3C-recombinant virus resulted in outgrowth and establishment of an LCL. This was unexpected because previous back-complementation experiments using similar RBPJ BM EBNA3C transfected into LCL with conditional EBNA3C, failed to rescue LCL proliferation after inactivation of the conditional EBNA3C [40,42]. Cell proliferation, measured by the incorporation of thymidine analogue EdU 36 days after primary B cell infection, showed that 22.9% of RBPJ BM EBNA3C cells were synthesising DNA, which is double the 11.7% of cells infected with EBNA3C KO virus, but considerably less than 55% of cells infected with wild type or revertant viruses (Fig 9B). Immunoblot analysis of the established RBPJ BM EBNA3C LCL 56 days after primary B cell infection showed similar EBNA3A, EBNA3B, EBNA3C, EBNALP and RBPJ levels compared with wild type or EBNA3C revertant LCLs (Fig 9C). EBNA2 expression, and probably as a consequence LMP1 expression, appeared to be significantly increased in the RBPJ BM EBNA3C LCL, even in comparison to EBNA3C knockout LCL. This suggests that the ability of EBNA3C to interact with RBPJ is important for the regulation of viral genes in the context of infection. The inability of RBPJ BM EBNA3C to bind to RBPJ was confirmed by immunoprecipitation of RBPJ from these LCLs. This very efficiently pulled down wild type EBNA3C, but only trace amounts of RBPJ BM EBNA3C (Fig 9D). RBPJ was immunoprecipitated efficiently from both LCLs.

**Fig 10:**
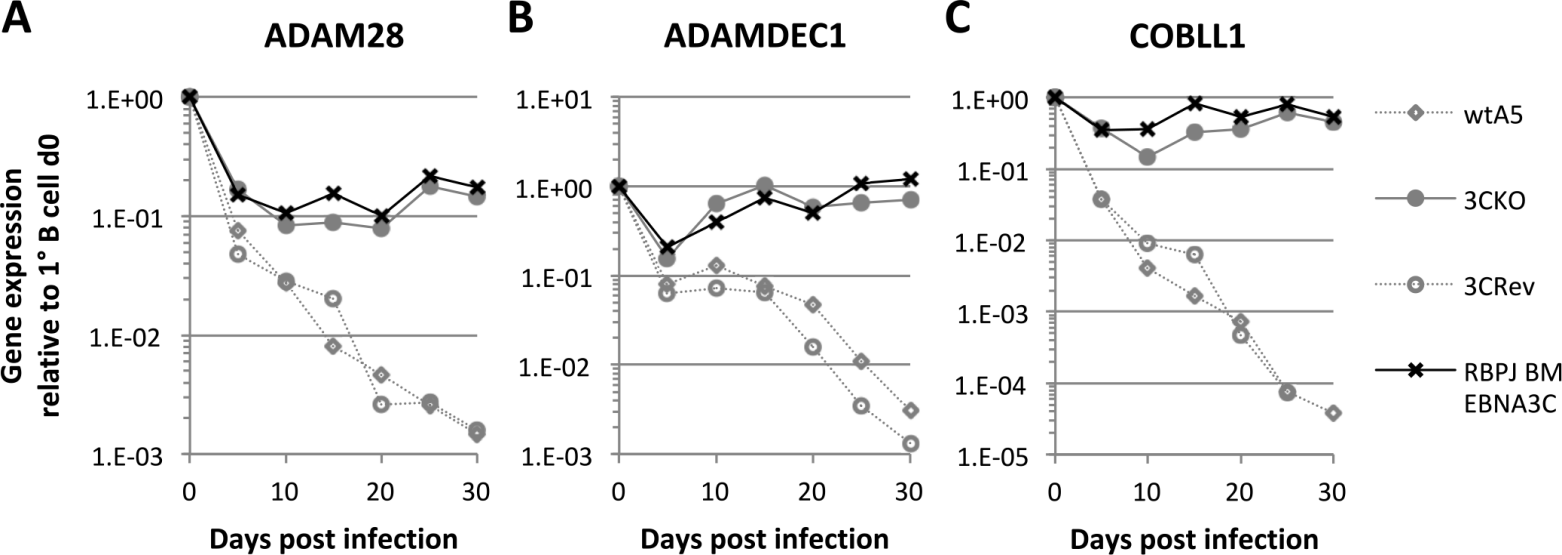
Ability of EBNA3C to bind to RBPJ appears to be essential for repression of *ADAM28*, *ADAMDEC1* and *COBLL1*. (**A-C**) Infection of primary B cell with either wild type (wtA5), EBNA3C knockout (3CKO), EBNA3C revertant (3CRev) or RBPJ binding mutant EBNA3C recombinant EBV. Gene expression of *ADAM28* (**A**), *ADAMDEC1* (**B**) and *COBLL1* (**C**) was normalised to *GNB2L1* and is shown relative to uninfected primary B cells.

Finally, in order to determine whether binding of EBNA3C to RBPJ was necessary for the repression of the endogenous *COBLL1* and *ADAM28-ADAMDEC1* locus, RNA samples taken every 5 days from the time of infection of primary B cells with the recombinant RBPJ BM EBNA3C virus were analysed. Consistent with the results of the luciferase reporter assays and the ChIP studies, infection with the RBPJ BM EBNA3C virus was unable to repress *ADAM28*, *ADAMDEC1* or *COBLL1* (Fig 10A–10C). Expression levels of all three genes were similar compared to changes seen after infection with EBNA3C KO virus, whereas infection with EBNA3C revertant or wild type viruses resulted in robust repression of all three genes as seen in the previous primary B cell infections (Fig 1). As before, these differences in gene expression between the various viruses were not seen for the control gene *ALAS1* (S1I Fig).

In conclusion, these results showed that the ability of EBNA3C to interact with RBPJ is not only essential for repression in transient reporter assays, but also for the repression of the endogenous *COBLL1* and *ADAM28-ADAMDEC1* locus in the context of viral infection.

## Discussion

Although it is well established that EBNA3C is essential for transformation of normal primary B cells and for repression of host tumour suppressor genes (*e*.*g*. *BCL2L11* and *CDKN2A*), the precise molecular mechanisms by which EBNA3C regulates gene expression remain largely unknown. Here, by using two genomic loci very robustly repressed by EBNA3C, we have explored some of the molecular interactions involved in EBNA3C-mediated gene repression. This has produced new insights into the temporal sequence of events during the repression and challenges existing models based on DNA-binding transcription factors remaining relatively static on chromatin.

Most surprising was the dynamic recruitment of and/or stabilisation of what we take to be RBPJ/EBNA3C complexes on both *COBLL1* and *ADAM* peaks. This is in contrast to the paradigm that RBPJ is stably bound to its DNA recognition sequences and that, in the absence of Notch signalling, recruits repressors that are replaced by activators upon signalling [13,61]. Furthermore, it is in contrast to previous reports that suggest EBNA3C disrupts RBPJ binding to DNA in order to prevent transactivation by EBNA2 [8,9,37]. It is tempting to speculate that interaction of EBNA3C with RBPJ increases the binding to–or stabilises RBPJ/EBNA3C complexes on–regulatory elements that control expression of these EBNA3C target genes. Recent studies in *Drosophila* [62] and mammalian cells [63] have revealed that activation of Notch signalling can induce *de novo* binding or increased binding of RBPJ mainly at regulatory elements. A similar observation was made for some EBNA2 target genes, where EBNA2 expression appeared to increase the occupancy of RBPJ at these genes during activation [64]. However, increased binding of RBPJ has not, to our knowledge, been reported during gene repression. It might be that EBNA3C, through interaction with one or more other transcription factors (S8 and S9 Figs), increases the on-rate in the dynamic equilibrium of RBPJ binding to its recognition sites. Using publically available transcription factor binding prediction software (PROMO [65,66] and Patch 1.0 [BIOBASE]), it was not possible to identify any strictly canonical RBPJ binding sites within 1kb around either the *ADAM* peak or the *COBLL1* peak, but if a more relaxed interpretation was used, several possible sites were found at both EBNA3C peaks. It is not possible to determine which, if any, of these are responsible for targeting RBPJ and it is more than likely that other transcription factors are also involved. Alternatively a combination of EBNA3C and some other factor could redirect RBPJ to cryptic sites. Previous studies found that EBNA3 binding sites coincided with various transcription factors, *e*.*g*. BATF, BCL11A, IRF4, PAX5 and RUNX3 [53,67,68], all of which seem to co-occupy the EBNA3 binding sites at *COBLL1* and *ADAM28/ADAMDEC1* (S8B and S9B Figs). Any of these could act as a co-factor in directing RBPJ/EBNA3C complexes to these particular loci.

This raises the question of precisely what role RBPJ plays in repression here, or more generally in the context of infections with other gamma-herpesviruses such as KSHV [43,44,69]. Our best guess is that the interaction between RBPJ and EBNA3C is needed for the assembly of a multi-protein platform of co-repressors (see Introduction and below) that is unable to efficiently assemble in the absence of either RBPJ or EBNA3C. It is possible that the assembly of these multi-protein complexes can mask the epitope detected by the anti-RBPJ antibody in ChIP experiments, which might be an explanation for what appeared to be lower RBPJ occupancy at *ADAM* peak and *COBLL1* peak at later time-points after activation of EBNA3C. Recently RBPJ has been found to be retained on mitotic chromatin–book-marking the transcriptional state of genes through cell division–and also to interact with CTCF, which might be involved in formation of higher-order chromosome structures [70]. We cannot exclude either of these functions being important here.

The functional importance of RBPJ for the repression of *ADAM28* and *COBLL1* was indicated in transient reporter assays using RBPJ BM EBNA3C and RBPJ knockout cells. In these assays EBNA3C binding appeared to convert enhancer-like elements into repressor elements. The magnitude of repression in these transient reporter assays was far less than the repression seen in the context of viral infection and the host chromosomes. Consequently we constructed the RBPJ BM EBNA3C recombinant virus–based on the most recent assessment of RBPJ binding sites in EBNA3C. The primary B cell infection with this virus unequivocally demonstrated that the RBPJ BM EBNA3C virus is completely unable to repress *ADAM28*, *ADAMDEC1* and *COBLL1*, providing compelling evidence that the EBNA3C:RBPJ interaction is essential in the context of viral infection. The only caveat here is that we cannot formally exclude the possibility that these two mutations in EBNA3C alter other yet to be described interactions required for gene repression. However, the failure of wild type EBNA3C to repress *ADAM28* luciferase constructs in the RBPJ-null DG75 cells also showed that presence of RBPJ is essential for repression. The RBPJ BM EBNA3C virus was able to establish a stable LCL (in culture >2 months) that, although it proliferated slowly, was in contrast to the previous back-complementation studies that suggested the interaction was essential to rescue LCLs when EBNA3C was inactivated [40–42]. Further studies are required to characterise the importance of the EBNA3C:RBPJ interaction and to determine which other factors are required for the dynamic binding of these complexes to specific regulatory elements in response to EBNA3C.

The repression of *ADAM28/ADAMDEC1* and *COBLL1* was highly reproducible and very similar between primary B cell infection and EBNA3C conditional LCL after activation of EBNA3C, which further validated the full functionality of the 3CHT fusion proteins. The kinetics followed a highly exponential repression profile over the first two weeks after infection or activation of EBNA3C (S3 Fig), but the fully repressed state was only achieved after 30 days or even later. Full de-repression, through inactivation of EBNA3C, required a similar time period. Currently, we do not understand why these changes in gene expression take place over such a long period of time. At least for *COBLL1* the initial repression was relatively rapid, with a 10-fold drop in gene expression by day three and a complete disappearance of COBLL1 protein in immunoblot by day six. However, reactivation of *COBLL1* expression and reappearance of COBLL1 protein from the fully repressed state took at least 20 days after inactivation of EBNA3C. One possible explanation is that it takes that long for repressive histone marks to be fully established during repression or fully removed during reactivation.

The comparison between histone marks and mRNA expression suggested that the initial repression of both loci is independent of polycomb protein recruitment, but requires removal of activation-associated histone acetylation and the H3K4me3 mark. There are 18 human proteins with deacetylase activity that are grouped into four families according to their homology (HDAC family 1–4) [71]. EBNA3C has been shown to bind to and recruit the class one HDAC family members 1 and 2, which makes both of them likely candidates involved in the initial repression [48,49]. There are more than 30 proteins in the Jumonji C family of demethylases that are able to remove mono-, di- or trimethylation on lysine residues and at least four of them, KDM5A/B/C/D, have been shown to be able to catalyse the removal of H3K4me3 [72–77]. Multiprotein complexes composed of both HDAC1 and HDAC2 together with KDM5A/Sin3 or KDM5C/NcoR/REST have been identified [76,78,79]. Besides HDAC1 and HDAC2, EBNA3C can bind to both Sin3, NcoR and CtBP [49,50], so it seems likely that EBNA3C recruits one of these multifunctional complexes to remove histone acetylation and H3K4me3 in the initial phase of repression. Furthermore RBPJ can independently recruit similar complexes of repressors (see Introduction), adding further support to the idea that by physically interacting EBNA3C and RBPJ synergise in the early phase of repression.

Following the initial repression, PRC1 and PRC2 were recruited probably to maintain or further extend the repressive state by depositing H3K27me3. In more recent studies the classical sequential recruitment model in which PRC2-induced modifications recruit PRC1 has been challenged. It has been shown that PRC1 can be recruited independently from PRC2 and the H3K27me3 modification [80–83]. Furthermore, PRC1 can actually recruit PRC2 through the deposition of H2AK119Ub [84–86]. In our study, however, the ChIP analysis revealed that–in contrast to the classical and more recent models of polycomb repressive complex recruitment–it appeared that PRC1 and PRC2 complexes were recruited not only in the same time frame, but also to different genomic loci. BMI1, as part of PRC1, was found at the EBNA3C binding site located in the regulatory elements of *ADAM28/ADAMDEC1* and *COBLL1*, whereas the PRC2 subunit SUZ12 was found at the TSS of *COBLL1*. No direct SUZ12 recruitment could be detected at various sites across the *ADAM28/ADAMDEC1* locus although H3K27me3 levels increased at these sites, albeit to much lower levels than at the TSS of *COBLL1*. Recruitment of SUZ12 to a discrete site might not have been detected because of the choice of primers, this is however unlikely, because a previous study in human embryonic stem cells identified that 95% of SUZ12 binding sites localised within 1kb of TSS and 40% were within 1kb of CpG islands [87]. Further studies verified this and showed that CpG islands could recruit PRC2 and led to the establishment of H3K27me3 [88–90]. The TSS of both *ADAM28* and *ADAMDEC1* were included in the ChIP analysis. Furthermore, there is a CpG island around the TSS of *COBLL1*, but not at the *ADAM28/ADAMDEC1* locus. This might explain the direct recruitment of SUZ12 and the much higher H3K27me3 levels at the TSS of *COBLL1* relative to the *ADAM28/ADAMDEC1* locus. It was very surprising that SUZ12 and BMI1 were recruited to two distinct sites at *COBLL1*. Polycomb complexes have been shown to mediate the formation of higher-order chromosome structures [91–93] (reviewed in [94]). So perhaps chromatin looping between the *COBLL1* peak and the TSS of *COBLL1* would bring BMI1 (PRC1) and SUZ12 (PRC2) together. We attempted chromosome conformation capture to analyse this locus but we could not reproducibly show looping between *COBLL1* peak and the TSS. We do not know the reason for this, however, the same technique has been successfully used to show repressive loop formation between *ADAM* peak and the TSS of *ADAM28* and *ADAMDEC1* when EBNA3C was expressed [53] and we have used it to show looping between a distal enhancer and TSS during EBNA3A/3C-mediated activation of a micro-RNA cluster [56].

It is currently unclear whether BMI1 and SUZ12 are recruited by direct interaction with EBNA3C or if this represents a default mechanism of gene repression at these two genomic loci once activation-associated histone marks are removed. A similar two-step model has been proposed recently for the EBNA3A-mediated repression of *CXCL9* and *CXCL10* [45]. At the *CXCL9/CXCL10* locus, EBNA3A binding to the regulatory sites displaced EBNA2 resulting in an initial state of repression (or de-activation), which was subsequently maintained or further extended by the recruitment of polycomb proteins. This is very similar to what we have observed, however, no occupancy of EBNA2 has been reported on the *ADAM* or *COBLL1* peaks (see below). Furthermore, it is currently unclear why EBNA3A plays only a subsidiary role in the regulation of *ADAM28* and *ADAMDEC1* compared to EBNA3C, or why EBNA3A is found on the *COBLL1* peak (S4 Fig) even though in the primary B cell infection the presence of EBNA3A is clearly not required in the repression of *COBLL1*. This is unlikely to be an artefact of our TAP-tagged LCL cell lines, because two recently published ChIP-seq experiments using anti-HA antibody in EBNA3A-HA [68] or EBNA3C-HA [67] LCL obtained similar results and detected both EBNA3A and EBNA3C at *ADAM* and *COBLL1* peaks (S8A and S9A Figs). Furthermore, confirming our RBPJ ChIP-qPCR results, ChIP-seq for RBPJ in the IB4 LCL [95] revealed steady-state occupancy of RBPJ on both sites (S8A and S9A Figs). Similarly, ChIP-seq in latency III expressing BL cell line MutuIII using the sheep polyclonal anti-EBNA3C antibody, which also precipitates EBNA3A and EBNA3B, identified the same peaks at both loci, but EBNA2 ChIP-seq revealed no binding at these loci ([53], S8B and S9B Figs).

It is worth noting that *RP11-624C23*.*1* –number four in the list of EBNA3C repressed genes found in the microarray transcriptome analysis (S1 Table)–is a long non-coding RNA with four isoforms of different lengths that run across the *ADAM28/ADAMDEC1* locus on the negative strand (S9B Fig). Together with *ADAM28* and *ADAMDEC1*, *RP11-624C23*.*1* is repressed by EBNA3C (http://www.epstein-barrvirus.org.uk), again consistent with the whole locus being co-ordinately regulated.

In summary, a detailed analysis of the mechanisms involved in EBNA3C-mediated gene repression of two genomic loci has provided novel insights into the temporal sequence of events during the repression of transcription and the dynamics of factor recruitment. First, it seems that histone marks associated with activation are removed in an initial step of the repression before repressive marks are deposited. Second, the sequential model of polycomb recruitment was not observed, but rather both PRC1 and PRC2 appeared to be recruited at the same time, but to different sites. Third, the paradigm that RBPJ is stably bound on DNA will need to be reassessed to accommodate a more dynamic recruitment and/or stabilisation model of RBPJ/EBNA3C complexes in repression, as has been proposed for RBPJ-mediated activation [62–64]. Although it is clearly essential, the role of RBPJ in EBNA3C-mediated repression examined here has still to be defined. Finally, the EBNA3C mutant incapable of binding RBPJ was able to sustain cell proliferation and to establish a stable LCL, suggesting a robust interaction between EBNA3C and RBPJ is not an absolute requirement for B cell proliferation.

## Material and Methods

### Primary B cell infection

Recombinant EBV KOs, Revs and wild type have been described previously [54]. Virus production and B cell isolation were performed as previously described [52,55]. Primary B cells were isolated from anonymous buffy coat residues (UK Blood Transfusion Service). B cell purity was assessed to be >90% CD20+ using anti-CD20-APC (eBioscience) staining and flow cytometric analysis. Three million primary B cells in 1.5 ml were infected with 0.5 ml of supernatant containing a total of 1–3x10^5^ Raji green units [55]. RNA from three million uninfected primary B cells was taken on the day of infection and infected cells were incubated in RPMI 1640 (Life Technologies) supplemented with 15% foetal bovine serum, penicillin, and streptomycin at 37°C and 5% CO2. For a period of 30 days, every three days—or five days for the second primary B cell infection with RBPJ binding mutant virus (see below) - 0.5 ml of cells were harvested for RNA extraction and replaced by fresh medium. After this, cells were, where possible, grown into LCL and analysed by immunoblotting.

### Cell culture and time-courses

All cells were cultured in RPMI 1640 medium (Life Technologies) supplemented with 10% foetal bovine serum, penicillin, and streptomycin either in absence or presence of 400nM 4-hydroxytamoxifen (HT, Sigma) at 37°C and 10% CO2. For the time-course experiments EBNA3C conditional LCLs (3CHT) on the p16-null background were used. These have been described previously [52,55]. 3CHT A13 (established in the absence of HT) were used in a time-course experiment over 60 days with samples taken for RNA, protein and ChIP every three days over the first 30 days and every ten days until day 60. Cells were counted and diluted to 3x10^5^ cells/ml at every time-point until day 30 and three times a week subsequently, but seeded at 3x10^5^ cells/ml the day prior to harvesting. After harvesting cells on day 30, half of the +HT culture was centrifuged and the medium replaced by fresh medium without HT and cultured without HT until day 60 (washed). 3CHT C19 (established in the presence of HT, washed and subsequently grown without HT in the medium for more than three months before the experiment was started) were used in a time-course experiment over 30 days. Cells were counted and split to 3x10^5^ cells/ml the day before harvesting samples for RNA, protein and ChIP.

### Reverse transcription quantitative PCR (RT-qPCR)

RT-qPCR was performed essentially as described previously [57]. RNA from 4.5x10^6^ cells was extracted using the RNeasy mini kit (Qiagen) and 10ng of cDNA was used for each qPCR reaction. *GAPDH* or *GNB2L1* were used as housekeeping genes as indicated and gene expression was expressed relative to primary B cells or LCL -HT on d0. The sequences of the primers used in this study are listed in S2 Table.

### Immunoblotting

Immunoblotting was performed essentially as described previously [54,55,57]. A total amount of 30 μg of RIPA protein extract was separated on 12, 10 or 7.5% SDS-PAGE, as appropriate, using a mini-PROTEAN II cell (BioRad) and transferred onto Protran nitrocellulose membrane. Antibodies used in this study are listed in S3 Table.

### Chromatin immunoprecipitation (ChIP)

ChIPs for histone modifications and SUZ12 were performed as described previously [57]. For anti-Flag, BMI1 and RBPJ ChIPs, 4.5x10^6^ cells were incubated for 20 min in 1 ml swelling buffer (25 mM HEPES pH 7.8, 1.5 mM MgCl_2_, 10 mM KCl, 0.1% NP-40, 1 mM DTT, 1 mM PMSF, 1 μg/ml aprotinin and 1 μg/ml pepstatin A). Nuclei were resuspended in 1 ml sonication buffer (50 mM HEPES pH 7.8, 140 mM NaCl, 1 mM EDTA, 1% Triton-X-100, 0.1% sodium deoxycholate, 0.1% SDS, 1 mM PMSF, 1 μg/ml aprotinin and 1 μg/ml pepstatin A) and sonicated for one hour using a Covaris M220 (75 W peak power, 26 duty cycle, 200 cycles/burst and 6°C set temperature). Thereafter, the ChIP assay kit from Millipore (17–295) was used, according to the manufacturer’s protocol. DNA was cleaned using QIAquick PCR purification Kit (Qiagen) and was assayed by qPCR on QuantStudio 7 Flex (Life technologies). Input DNA was 5% of DNA used in immunoprecipitations and diluted to 2.5% prior to PCR quantification. Enrichment relative to input was calculated using four 5-fold-dilution series and error bars calculated as standard deviations from triplicate PCR reactions for both input and IP. Antibodies used are listed in S3 Table and sequences of the primers used in these assays are listed in S4 Table.

### Transient reporter assays

Genomic DNA extracted from GM12878 LCL cells using Blood & Cell Culture DNA Midi kit (Qiagen) was used to PCR amplify the promoter region 1 kb upstream of *ADAM28* and *COBLL1* (short transcripts), the *ADAM* peak (1 kb around the EBNA3 binding peak at *ADAM28*-*ADAMDEC1*) or the *COBLL1* peak (1.5 kb around the EBNA3 binding peak at COBLL1) (Primers are listed in S5 Table). The promoter regions were cloned upstream of the luciferase gene in pGL3-basic vector using the MluI restriction site. ADAM peak and COBLL1 peak were cloned downstream of the luciferase gene using the SalI restriction site. All vectors were screened for the correct orientation and were sequence verified. DG75 (for *ADAM28* constructs) or Raji cells (for *COBLL1* constructs) were electroporated with 1 μg of pGL3-luciferase vectors, 1 μg pSV-beta-galactosidase and varying amounts of pCDNA3-EBNA3 expression plasmids. Total amounts of DNA were balanced using an empty pCDNA3 expression plasmid. Electroporations were performed as described previously for DG75 [96]. For Raji a voltage of 240V was used and all electroporations were harvested after 48h. Luciferase and beta-galactosidase assays were performed as described previously [96] and measured on FLUOstar Omega (BMG Labtech). Beta-galactosidase activity was used to normalise luciferase activities for transfection efficiencies.

### Virus construction

For the creation of the RBPJ binding mutant (BM) EBNA3C recombinant virus, the N terminus of EBNA3C was cloned from the B95.8 EBV-BAC [97] by XbaI digestion at position 98,398 and BglII at site 99,749 (relative to GenBank entry V01555.2) into a modified pBlueScrit II SK+. The two in previous studies identified RBPJ binding site of EBN3C were mutated to generate the RBPJ binding mutant EBNA3C. In-Fusion PCR mutagenesis (Clonetech) was employed to first substitute EBNA3C residues _209_TFGC for _209_AAAA while introducing a NotI recognition site (forward primer: 5’-GCGGCCGCAGCTCAAAATGCGGCACGAACT-3’, reverse primer: 5’-AGCTGCGGCCGCGGCAGTTAACATGATGCTGT-3’). PCR products were purified (Diffinity RapidTip2) and circularised using In-Fusion cloning (Clonetech). Plasmid DNA was screened by NotI restriction digest before introducing the W227S mutation together with a SalI recognition site (forward primer: 5’-GCCACCGTGTCGACACCACCCCATGCTGGACCAA-3’, reverse primer: 5’-CGACACGGTGGCAGAGAAGGTGT-3’). The RBPJ binding mutant fragment of EBNA3C was subcloned into the shuttle plasmid pKovKanΔCm [98] and verified by DNA sequencing. The recombinant EBV was created by RecA based homologous recombination between the B95.8 EBV-BAC and the shuttle plasmid as previously described [98]. At each stage of recombineering BAC DNA was isolated and validated by restriction digest and pulsed-field gel electrophoresis. RBPJ binding mutant EBNA3C virus producing 293 cell clones were established as previously described [54]. Episome rescue of EBV BACs from 293 producing cell lines was performed as previously described for low molecular weight DNA [99].

### Immunoprecipitation (IP)

IPs were performed essentially as described previously [96]. Briefly, RBPJ was immunoprecipitated from protein extracts of 10^7^ wild type EBNA3C or RBPJ BM EBNA3C LCLs for two hours at 4°C using RBPJ rat monoclonal antibody 1F1. Then, 30 μl of protein G-Sepharose beads were added and incubated under rotation for 1h at 4°C, washed four times in IP buffer and immunoprecipitated proteins were resolved by SDS-PAGE and probed for EBNA3C (A10) or RBPJ (ab25949).

### Cell proliferation assay

A cell proliferation assay–based on measuring the incorporation of EdU at day 36 after primary B cell infection–was performed as described previously for established cell lines [52].

## Supporting Information

S1 Fig (A-B) Infection of primary B cells with wild type (wtI6) or recombinant EBNA3 knockout mutant (KO) or revertant (Rev) EBV. Gene expression for *ALAS1* (A) and *GNB2L1* (B) was normalised to *GAPDH* and is shown relative to uninfected primary B cells. (C-E) LCL 3CHT A13 or LCL 3CKO were grown for 60 days either in the absence (-HT) or presence (+HT) of HT. Gene expression for *ADAM28* (C), *ADAMDEC1* (D) and *COBLL1* (E) was normalised to *GAPDH* and is shown relative to 3CHT -HT on d0. (F-H) Time-course using EBNA3C-conditional LCL 3CHT C19. Cells were grown over 30 days either in absence of HT (-HT) or presence of HT (+HT). Gene expression for *ADAM28* (F), *ADAMDEC1* (G) and *COBLL1* (H) was normalised to *GAPDH* and shown relative to -HT on day 0. (I) Infection of primary B cells with wild type (wtA5), EBNA3C knockout (3CKO), EBNA3C revertant (3CRev), or RBPJ binding mutant EBNA3C recombinant EBV. Gene expression for *ALAS1* was normalised to *GNB2L1* and is shown relative to uninfected primary B cells.
(EPS)

S2 Fig (A) Immunoblot for COBLL1, EBNA3A, EBNA3B, EBNA3C, p16, Retinoblastoma protein (Rb), Phospho-Rb and γ-tubulin on wild type, EBNA3 knockout (KO) or revertant (Rev) LCLs grown out from primary B infection. (B) Immunoblot for COBLL1, EBNA3A, EBNA3B, EBNA3C, SUZ12, BMI1, RBPJ and γ-tubulin on 3CHT A13 time course grown over 60 days either in absence of HT (-HT), presence of HT (+HT) or without HT after 30 days +HT for 10, 20, and 30 days (washed). (C) As in (B) but on 3CHT C19 time course. Black arrows indicate COBLL1 band and non-specific band (NS). (D) Immunoblot for RBPJ and γ-tubulin on DG75 lacking RBPJ (DG75ΔRBPJ) and RBPJ competent DG75. (EPS)

S3 Fig Kinetics of EBNA3C-mediated gene repression. Kinetics of *COBLL1* (**A**), *ADAM28* (**B**) and *ADAMDEC1* (**C**) repression after infection of primary B cells with wild type (wtI6) EBV or after activation of EBNA3C in 3CHT A13 and C19 LCLs in two replicate time-courses each (Rep1+2). Gene expression was normalised to *GAPDH* and expressed relative to -HT d0 for A13 and C19 3CHT or uninfected primary B cells for wtI6. Exponential trendlines were fitted and equations and R^2^ values are shown.
(EPS)

S4 Fig Validation of EBNA3A and EBNA3C binding at *ADAM* peak and *COBLL1* peak by ChIP-qPCR. Anti-Flag ChIP was performed on wild type (WT), tandem affinity purification (TAP) tagged EBNA3A (EBNA3A-TAP) or EBNA3C (EBNA3C-TAP) LCLs. ChIP values represent enrichment at *myoglobin* (MyoG), *ADAM* peak or *COBLL1* peak relative to input ± standard deviations of triplicate qPCR reactions for ChIP and input of each sample.
(EPS)

S5 Fig Epigenetic changes and factor recruitment to *COBLL1* locus during 3CHT C19 time-course. (**A-G**) ChIP for H3K9ac (**A**), H3K27ac (**B**), H3K4me3 (**C**), H3K27me3 (**D**), SUZ12 (**E**), BMI1 (**F**) and RBPJ (**G**) on samples from 3CHT C19 time-course at locations within the *COBLL1* locus (see Fig 4), at *GAPDH* or *myoglobin* as indicated. Cells were grown in the absence (-HT) or presence of HT (+HT) and numbers indicate the day of harvest. ChIP values represent enrichment relative to input ± standard deviations of triplicate qPCR reactions for ChIP and input of each sample.
(EPS)

S6 Fig Epigenetic changes and factor recruitment to *ADAM28-ADAMDEC1* locus during 3CHT C19 time-course. (**A-F**) ChIP for H3K9ac (**A**), H3K27ac (**B**), H3K4me3 (**C**), H3K27me3 (**D**), BMI1 (**E**) and RBPJ (**F**) on samples from 3CHT C19 time-course at locations across the *ADAM28-ADAMDEC1* locus (see Fig 5), at *GAPDH* or *myoglobin* as indicated. Cells were grown in the absence (-HT) or presence of HT (+HT) and numbers indicate the day of harvest. ChIP values represent enrichment relative to input ± standard deviations of triplicate qPCR reactions for ChIP and input of each sample.
(EPS)

S7 Fig Validation of successful RBPJ BM EBNA3C recombinant EBV BAC creation. (**A**) BACs of wild type (WT) and newly created RBPJ binding mutant EBNA3C (BM) EBV were analysed by restriction digestion and pulsed-field gel electrophoresis. NotI restriction digestion showed introduction of the _209_AAAA mutation that created an additional NotI restriction site that cut the 36,807 bp wild type band into two bands of 18,540 bp and 18,267 bp. SalI restriction digestion showed introduction of the W227S mutation that created an additional SalI restriction site that cut the wild type 29,695 bp band into a 23,521bp and 6,174bp band. EcoRI and AgeI restriction enzyme digestion revealed that the integrity of the BAC has been maintained during the recombination process when compared to the WT EBV BAC. (**B**) Episomes were rescued from HEK 293 virus producing cell lines for six colonies of the mutant (named A-F) and compared to WT EBV. BACs were analysed as in (A). All colonies except E show the expected banding pattern.
(EPS)

S8 Fig EBNA3A and EBNA3C binding sites on *COBLL1* locus are co-occupied by multiple transcription factors. (**A**) UCSC genome browser overview of *COBLL1* genomic locus showing ChIP-seq tracks for RBPJ [95], EBNA3A-HA [68] and EBNA3C-HA [67] aligned to hg18. Occupancy levels are merely to indicate the position and are not to scale. (**B**) Hg19 view of same genomic locus as in (**A**) showing ChIP-seq tracks for EBNA2 [53], EBNA3 [53], EBNA3A-TAP, EBNA3C-TAP, the TSS (horizontal arrow), ENCODE ChIP-seq tracks for H3K4me3 and H3K27ac in CD20^+^ primary B cells (black) or LCL GM12878 (red) and ENCODE ChIP-seq peaks for various transcription factors in GM12878 LCL. The EBNA3A and EBNA3C binding site is highlighted by inclusion in a dashed box.
(EPS)

S9 Fig EBNA3A and EBNA3C binding sites on *ADAM28/ADAMDEC1* locus are co-occupied by multiple transcription factors. (**A**) UCSC genome browser overview of *ADAM28/ADAMDEC1* genomic locus showing ChIP-seq tracks for RBPJ [95], EBNA3A-HA [68] and EBNA3C-HA [67] aligned to hg18. Occupancy levels are merely to indicate the position and are not to scale. (**B**) Hg19 view of same genomic locus as in (**A**) showing ChIP-seq tracks for EBNA2 [53], EBNA3 [53], EBNA3A-TAP, EBNA3C-TAP, the TSSs of *ADAM28* and *ADAMDEC1* (horizontal arrows) on the positive strand (left to right), long non-coding RNA RP11-624C23.1 on the negative strand (right to left), ENCODE ChIP-seq tracks for H3K4me3 and H3K27ac in CD20^+^ primary B cells (black) or LCL GM12878 (red) and ENCODE ChIP-seq peaks for various transcription factors in GM12878 LCL. The EBNA3A and EBNA3C binding site is highlighted by inclusion in a dashed box.
(EPS)

S1 Table List of top 10 repressed genes by EBNA3C identified by microarray transcriptome analysis [52]. (EPS)

S2 Table Gene expression RT-qPCR primer sequences. (EPS)

S3 Table List of antibodies. (EPS)

S4 Table ChIP qPCR primer sequences. (EPS)

S5 Table Cloning primers for transient reporter vectors. (EPS)
